# Does Adhesive Luting Reinforce the Mechanical Properties of Dental Ceramics Used as Restorative Materials? A Systematic Review and Meta-Analysis

**DOI:** 10.3290/j.jad.b2916469

**Published:** 2022-04-13

**Authors:** Lucas Saldanha da Rosa, Kiara Serafini Dapieve, Fernanda Dalla-Nora, Marília Pivetta Rippe, Luiz Felipe Valandro, Rafael Sarkis-Onofre, Gabriel Kalil Rocha Pereira

**Affiliations:** a PhD Student, Postgraduate Program in Oral Sciences, Federal University of Santa Maria (UFSM), Santa Maria, RS, Brazil. Study concept and design, acquisition and analysis of data, interpretation of data, wrote and reviewed the manuscript.; b PhD Student, Postgraduate Program in Oral Sciences, Federal University of Santa Maria (UFSM), Santa Maria, RS, Brazil. Acquisition and analysis of data, interpretation of data, wrote and reviewed the manuscript. Study concept and design, wrote and reviewed the manuscript.; c Associate Professor, Postgraduate Program in Oral Sciences, Federal University of Santa Maria (UFSM), Santa Maria, RS, Brazil. Study concept and design, wrote and reviewed the manuscript.; d Professor, Postgraduate Program in Oral Sciences, Federal University of Santa Maria (UFSM), Santa Maria, RS, Brazil. Study concept and design, wrote and reviewed the manuscript.; e Professor, Graduate Program in Dentistry, Meriodional Faculty (IMED), Passo Fundo, Brazil. Data analysis and interpretation, reviewed the manuscript.; f Adjunct Professor, Postgraduate Program in Oral Sciences, Federal University of Santa Maria (UFSM), Santa Maria, RS, Brazil. Study concept and design, acquisition and analysis of data, interpretation of data, wrote and reviewed the manuscript.

**Keywords:** adhesion, cementation technique, dental ceramic, luting, mechanical property

## Abstract

**Purpose:**

This systematic review aims to explore and compile the effect of adhesive luting on the mechanical properties of dental ceramics used as restorative materials.

**Materials and Methods:**

The PubMed/MEDLINE, Web of Science and Scopus databases were searched on January 31st, 2021 to select laboratory studies written in English, without publishing-date restrictions, which compared the mechanical properties of commercially available dental ceramics as restorative materials luted using adhesive vs non-adhesive strategies. A total of 20 (out of 2039) studies were eligible and included in the analysis. Two authors independently selected the studies, extracted the data and assessed the risk of bias. Mean differences (RevMan5.1, random effects model, α = 0.05) were obtained by comparing resistance values of adhesive and non-adhesive conditions (global analysis). Subgroup analyses were performed considering ceramic composition and aging.

**Results:**

In the global analysis, adhesive luting induced higher mechanical resistance values compared to non-adhesive luting (p ≤ 0.01). The same effect was observed for glass and alumina ceramics (p ≤ 0.01), but not for zirconia polycrystals (p = 0.83). Adhesive luting was favorable in both the aged and non-aged subgroup analysis (p ≤ 0.01). High heterogeneity was found in all meta-analyses. All analyzed studies in the systematic review scored negatively for risk of bias in most of the factors considered.

**Conclusions:**

Adhesive luting reinforces the mechanical properties of dental ceramics used as restorative materials, with the exception of zirconia polycrystals.

Indirect ceramic restorations have been widely used in dentistry, especially due to their superior esthetic and mechanical properties when compared to resin composites.^22^ The failure of these restorations can be attributed to a series of factors, such as residual stresses, contact damage, presence of defects as pores, microcracks, regions with loss of bonding that may predispose stress concentration, and crack growth during mechanical loading.^1,46,79,91^

Cements which do not benefit from adhesion mechanisms between the ceramic and the cementation agent, such as traditional glass-ionomer and zinc-phosphate cements, only rely on mechanical interlocking to achieve bonding.^29^ Alternatively, most protocols for resin cements have adhesive properties which can be considered as mechanisms that go beyond traditional bonding based solely on the retention offered by conventional acid-base cement systems.^29^ In addition, resin cements adapt better to the restoration’s margins and minimize marginal leakage,^34^ even though they have more sensitive application protocols owing to multiple steps and moisture control.^51^

In this regard, the gold standard bonding protocol for glass ceramics has been hydrofluoric acid etching and subsequent application of a coupling agent.^15,26^ While the acid selectively dissolves the glassy matrix, the silane coupling agents (bifunctional molecules which act as a link between the organic phase of the resin cement and the silica present in glass ceramics) confer the chemical bonding characteristic.^52^ This interaction generates a consistent unit that provides great stress distribution over the restoration, improving its mechanical properties.^41,45,53^ In addition, the use of adhesive materials conforms to the concepts of minimally invasive dentistry,^51^ as adhesive luting interacts with ceramic surfaces and promotes crack bridging.^87^ In this situation, silane molecules inside the cracks, together with resin cement shrinkage during polymerization, make crack opening and spreading difficult.^89^

The concepts for polycrystalline ceramics are different, since the absence of a glassy phase prevents them from being etched by the conventionally-used acids.^60^ This situation requires application of a tribochemical silica coating as a surface treatment.^5,14,18,80^ Thus, after applying a thin layer of silicon oxide via air abrasion with silica-coated alumina particles, the same silane coupling agent used for glass ceramics is used here to promote chemical bonding.^52^ Another technique used is air abrasion with aluminum oxide alone. This method relies on the microretentions generated by the impact of the aluminum particles on the ceramic surface^18,56^ and the chemical interaction with 10-methacryloyloxydecyl dihydrogen phosphate (10-MDP) present in some adhesive materials and the hydroxyl groups present in zirconia.^54,56,59^

Despite the assumptions mentioned above, a study to synthesize all existing data in this regard is still required to generate high-quality scientific knowledge to corroborate the importance of adhesive luting for reinforcing the mechanical properties of dental ceramics. Therefore, a systematic review that compiles all existing in vitro data about this topic and organizes it through a meta-analysis may help to answer this research question, and could be an important contribution towards understanding the relation between ceramics, cements, and stress distribution in indirect restorations. Its results would support clinical decision-making with the best evidence-based practice. Thus, the aim of this systematic review and meta-analysis was to explore and compile the effect of adhesive luting on the mechanical properties of dental ceramics used as restorative materials.

## Materials and Methods

This systematic review was reported according to the PRISMA 2020 statement.^61^

The following research question was formulated to address the literature and outline the search strategy: Does adhesive luting reinforce the mechanical properties of dental ceramics used as restorative materials?

### Registration and Selection Criteria

The protocol of this study was made available online (https://osf.io/vtnjk/).

#### Inclusion criteria

We selected studies in dentistry which considered the mechanical properties of all dental ceramics used as restorative materials that were cemented using adhesive and non-adhesive strategies. Studies that compared the effect of at least one adhesive luting strategy vs a non-adhesive strategy were included. Two subgroups were considered: one in which the systems classically known as adhesive (ie, resin cements) were compared with systems classically known as non-adhesive (ie, zinc-phosphate cement, glass-ionomer cement); and the other in which studies that used the same system in adhesive and non-adhesive approaches were compared (ie, substrate isolated with some agent or not), regardless of the ceramic used (eg, feldspathic, leucite, lithium disilicate, lithium silicate, alumina, zirconia, among others), the processing method for ceramic manufacturing (layering, pressing, or CAD/CAM techniques, etc), the mechanical property measured (strength, hardness, toughness, etc), and regardless of the testing method (monotonic, fatigue, etc). In terms of the study designs and based on the outcomes considered, only in vitro studies were included.

#### Exclusion criteria

We excluded studies in dentistry that did not compare adhesive vs non-adhesive luting strategies/systems, did not use a tooth substrate (human or animal) or a validated tooth analogue, were not written in English, and did not use a commercially available ceramic.

### Search

The search was last performed on January 31st, 2021, in three databases: MEDLINE via PubMed, Web of Science, and Scopus, limited to articles written in English, without publishing-date restrictions. The search strategy (Table 1) was based on MESH terms and free-text specific terms of PubMed, which were adapted for the other databases, if necessary.

**Table 1:** Search strategy

PUBMED – 1428 results
((((ceramic) or (glass-ceramics) or (porcelain) or (leucite) or (feldspathic) or (lithium disilicate) or (lithium silicate) or (polycrystalline) or (zirconia) or (alumina) or (yttrium stabilized zirconia) or (YSZ) or (Y-TZP) or (YPSZ)) and ((cementation) or (luting)) and ((mechanical properties) or (failure load) or (strength) or (resistance) or (compression) or (fracture) or (retention) or (tensile)) and ((in vitro) or (laboratorial) or (in lab))))
WEB OF SCIENCE – 563 results
#1 TS=(ceramic or glass-ceramics or porcelain or leucite or feldspathic or lithium disilicate or lithium silicate) #2 TS=(cementation or luting) #3 TS=(mechanical properties or failure load or strength or resistance or compression or fracture or retention or tensile) #4 TS=(in vitro or laboratorial or in lab) #5 #4 AND #3 AND #2 AND #1
SCOPUS – 1554 results
(“ceramic” or “glass-ceramics” or “porcelain” or “leucite” or “feldspathic” or “lithium disilicate” or “lithium silicate”) and (“cementation” or “luting”) and (“mechanical properties” or “failure load” or “strength” or “resistance” or “compression” or “fracture” or “retention” or “tensile”) and (“in vitro” or “laboratorial” or “in lab”) and not (“review”)

### Screening

The search was initially undertaken using Rayyan QCRI, an online platform for systematic reviews.^58^ Two researchers independently identified the articles by first analyzing titles and abstracts for the presence of the eligibility criteria. Retrieved records were classified as include, exclude, or uncertain. The full-text articles of the included and uncertain records were selected for further eligibility screening independently by the same 2 reviewers. Discrepancies in screening of titles/abstracts and full text articles were resolved through discussion. In case of disagreement, the opinion of a third reviewer was solicited.

### Data Extraction

Two researchers independently extracted the data to a form using Excel. After extraction, the results were checked by both researchers to be certain of the data. Any disagreement was solved by discussion until a consensus was reached.^59^ Then, two reviewers independently extracted the data and another checked it. The following data were collected: study design; characteristics of ceramic material used; luting system and cementation method; type of substrate; restoration geometry (anatomic crown, simplified crown, simplified restoration); aging characteristics; mechanical property measured (outcomes) and the results (mean and standard deviation), testing method; and main findings of the study.

### Risk of Bias Assessment

The risk of bias assessment was performed independently by two researchers, based on and adapted from a previous study.^71^ After checking the assessment made by each researcher, any disagreement was solved by discussion until consensus was attained. The following parameters for the study’s quality assessment were considered: sample size estimation, randomization of ceramic specimen, sintering/crystallization cycle used according to the manufacturer’s instructions, specimen preparation clearly stated and executed in a standardized and reproducible manner, test design and outcome in accordance with international standard rules (ie, ISO, ASTM, and others), cementation protocol clearly specified, test executed by a single blinded operator, and the presence of fractographic/failure analysis. Graphics were performed in the Review Manager 5.1 software program (Nordic Cochrane Center, Cochrane Collaboration; Copenhagen, Denmark).

### Data Analysis

A descriptive analysis was performed considering the collected main characteristics of the studies. A meta-analysis was conducted in the Review Manager 5.1 software program (Cochrane Collaboration) using a random effect model considering the evaluated outcomes (mechanical properties). Pooled effect estimates were obtained by comparing raw mean differences among conditions for each outcome and sub-grouped by ceramic type, cementation methods, and aging. Negative estimates favored adhesive luting. p<0.05 was considered statistically significant (Z test). Statistical heterogeneity among studies was assessed via the Cochran Q test, with a threshold p-value of 0.1, and the inconsistency test I^2^, in which values higher than 50% were considered indicative of high heterogeneity.

## Results

### Descriptive Analysis

**Fig 1:**
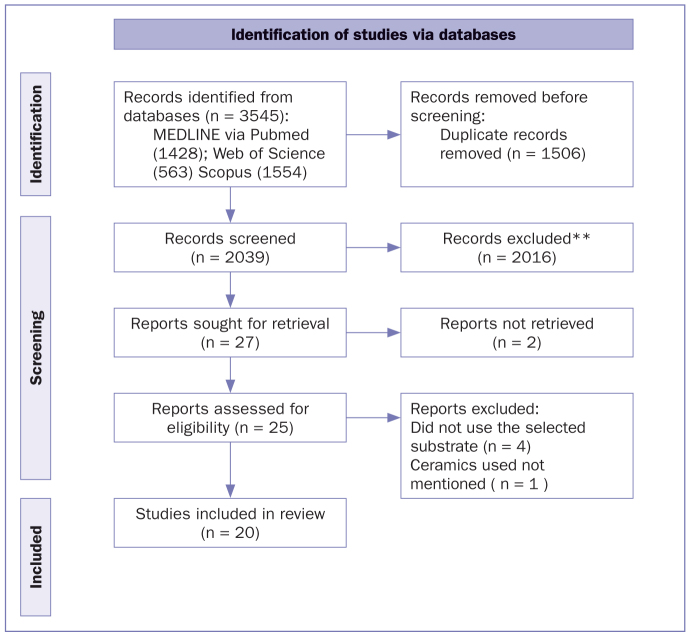
Flow diagram illustrating the identification of studies via databases, and the screening steps for the selection of studies, in accordance with the PRISMA 2020 statement.^61^

We analyzed 25 studies, four of which did not use the selected substrates.^25,62,75,88^ One did not mention the ceramics used,^20^ and we could not obtain the information from the author. Thus, these five studies were excluded, and the remaining 20 studies were considered (Fig 1).

Characteristics of the studies are summarized in Table 2. Regarding the restorative materials, most of the articles (11) used glass ceramics,^4,9,17,30,37,41,42,53,55,66,85^ six used alumina,^4,12,42,55,69,72^ and four used zirconia.^19,57,68,70^ All studies that worked with zirconia used it with a monolithic design. Some studies investigated more than one ceramic type. The majority of the studies^16^ used human teeth, and four studies used glass-fiber–filled epoxy resin as dentin analogue. In terms of restorations designs, fourteen used anatomic crowns, three used simplified crowns, two used simplified restorations, one used inlays, and one used full anatomic three-element bridges. Only two studies used a resin cement applied with prior substrate isolation to create a non-bonding scenario, while the majority of the studies^18^ compared conventional cementation systems to adhesive cements.

**Table 2:** Characteristics of included studies

Study	Study design	Ceramic material used	Luting system and cementation method	Type of substrate	Restoration geometry	Aging	Mechanical property measured	Testing method
Jensen et al, 1989	Anterior and posterior crowns luted with GIC or filled luting resin. n= 5	Porcelain	Crowns: Etched (not specified). Enamel etched with orthophosphoric acid for 1 min. Glass ionomer: Ketac Bond. Dentin etched with 10% polyacrylic acid. Apply cement. Resin bonding Crown: silane Teeth: bonding agent (Scotchbond 2 layers, Gluma 1 layer). Apply filled luting resin.	Sound and fresh extracted teeth	Anatomic anterior and posterior crowns	Thermal cycles: 500 times between 5°C 55°C, 30 s in each water bath	Fracture resistance (Kgf)	Load applied parallel to the long axis of the tooth with a 3-mm diameter hemisphere at 0.05 cm/min until catastrophic failure
Dietschi et al, 1990	Vitadur N/compisite resin cement; Vitadur N/GIC; Ceramco II/compisite resin cement; Ceramco II/GIC. n= 10	Vitadur N: feldspathic powder/liquid ceramic Ceramco II: feldspathic powder/liquid ceramic	Dicor: chemical and light-curing composite resin (inlays: 40% HF 3 min; tooth: 37% H_3_PO_4_ 60 s) Aqua-Cem: glass-ionomer luting cement	Human teeth	Inlay/ Veneer	Absent	Fracture load (kgf)	Compression; 2-mm–diameter ball; 1 mm/min
McCormick et al, 1993	Hi-Ceram Biomer; Hi-Ceram ZnPO_4_, Hi-Ceram Ketak-Cem; Dicor Biomer; Dicor ZnPO_4_, Dicor Ketak-Cem; natural teeth. n= 10	Hi-Ceram: alumina-reinforced felspathic ceramic Dicor: glass ceramic	Zinc-phosphate cement (Fleck’s, Mizzy), glass-ionomer cement (Ketac-Cem, 3M Oral Care), autopolymerizing composite resin cement (Biomer, Caulk/Dentsply)	Human teeth	Anatomic crown	Absent	Fracture load (kgf)	Compression with a 4-mm steel ball at 0.5 mm/min until fracture.
Burke, 1995	Group 1: ceramic etched and silanized, dentinal bonding procedures, resin composite cement Group 2: ceramic not etched and silanized, dentinal bonding procedures, resin composite cement Group 3: ceramic etched and silanized, no dentinal bonding procedures, resin composite cement Group 4: ceramic not etched and silanized, not dentinal bonding procedures, phosphate cement n= 10	Mirage fiber porcelain: feldspathic powder/liquid ceramic	Mirage ABC/FLC kits: tooth conditioning and primer application. Dual-curing. Light for 40 s. Zinc oxyphosphate cement: conventional application	Human teeth	Anatomic crown	Absent	Fracture load (N)	Compression with a 4-mm ball; 1 mm/min
Scherrer et al, 1996	Comparative groups of intact extracted molar and three types of crowns: feldspathic porcelain (luted with zinc-phosphate and luted with resin cement), glass-ceramic (resin cement), and glass-infiltrated alumina (resin cement). n=10.	Feldspathic porcelain (Ceramco conventional: powder and liquid), glass-ceramic (Dicor), and glass-infiltrated alumina (In-Ceram)	Zinc-phosphate cement (without previous treatment). Resin cement (Dicor light-activation kit, Dentsply). Acid etching and silanization of ceramic, dentin pretreated using a primer and adhesive (Prisma Universal Bond, De Trey/Dentsply)	Human teeth	Simplified anatomy crown	Absent	Fracture resistance (N)	Compression; 12.7 mm in diameter ball contacted the crown at three distinct points at a crosshead speed of 0.5 mm/min
Leevailoj et al, 1998	In-Ceram (Fuji I; Fuji Plus; Vitremer; Advance; Panavia 21) Feldspathic (Fuji I; Fuji Plus; Vitremer; Advance; Panavia 21) stored in NaCl unitl 2 months. After, the survivors were tested for fracture strength. n= 10	In-Ceram 0.5 mm core + Vitadur Alpha (powder/liquid feldspathic) 1.5 mm Vitadur Alpha 0.5 mm core (porcelain) + Vitadur Alpha 1.5 mm	Fuji I: conventional glass-ionomer cement Fuji Plus: resin-modified glass-ionomer cement. Fuji Plus conditioner on tooth for 20s Vitremer: resin-modified glass-ionomer cement Advance: fluoride-releasing resin cement. PENTA primer on tooth Panavia 21: resin cement. Apply ED primer A and B on tooth. Panavia etching agent 5s + Clearfil New on crowns.	Human teeth	Simplified crown	37°C in 0.8% NaCl solution at 1 h, 6 h, 24 h, 2 days, 3 days, 1 week, 2 weeks, 3 weeks, 1 month, and 2 months	Fracture load (N)	Compression; 3.2-mm–diameter ball; 0.5 mm/min
Behr et al, 2003	Carrara/Variolink II; Carrara/Fuji Plus; Carrara/Temp Bond. n= 8	Carrara press - Leucite-reinforced ceramic (press)	Variolink II: low viscosity, dual-curing/light-curing resin based dental luting material; 35% H_3_PO_4_, primer 15 s, adhesive 15 s, cement Fuji Plus: radiopaque reinforced glass-ionomer luting cement, liquid/powder self-curing material; citric acid, cement Temp Bond: zinc oxide-eugenol cement +A1:M4nt - self-curing material; cement	Human teeth	Anatomic crown	1,200,000 cycles; 1.66 Hz; 50 N; no piston reported + 6000 thermal cycles	Fracture load (N)	Compression; 4-mm–diameter ball; 1 mm/min
Okutan et al, 2006	ZrSiO4 crowns cemented with KetacCem or Panavia 21EX. n= 16	KaVo Everest HPC ZrSiO_4_ CAD/CAM ceramic	The inner surfaces of all of the crowns were sandblasted before cementation was performed. Glass-ionomer cement: KetacCem conventional GIC Autopolymerizing composite cement (containing 10-MDP): Panavia 21EX: etching agent and ED Primer on tooth + cement	Human teeth	Anatomic crowns	1,200,000 cycles; 1.3 Hz; 49 N; 6-mm diameter ceramic antagonist ball; thermocycling 5°C–55°C for 60 s each	Fracture load (N)	Compression load was applied to the occlusal surface of samples at 2 mm/min.
Attia et al, 2006	Ceramic (Vita Mark II) and millable composite resin crowns (MZ100 Block) were fabricated using a CAD/CAM system and cemented with 3 luting agents: RelyX ARC (RX), GC Fuji CEM (FC), and zinc-phosphate cement (ZP). n=16	CAD/CAM feldspathic ceramic (Vita Mark II)	All crowns were etched using 4.9% HF for 1 min Group 1: RelyX ARC (dual-polymerizing resin cement). Prepare teeth with 37% H_3_PO_4_ and Single Bond Group 2: GC Fuji CEM, prepare teeth with conditioner Group 3: zinc-phosphate cement	Human teeth	Anatomic crowns	Half of the specimens in each subgroup (n=8) were “fatigued” in a masticatory simulator (600,000 masticatory cycles and 3500 thermal cycles). 1.2 Hz, maximum load 49 N, minimum load 0 N, and lateral component 0.3 mm. Steatite ceramic balls (4-mm diameter) were used as antagonistic surfaces to simulate the antagonist teeth	Fracture load (N)	Compression; 4-mm-diameter ball; 1 mm/min
Blatz et al, 2008	Zinc-phosphate cement without any pretreatment; universal adhesive resin cement without any pretreatment; composite resin containing adhesive phosphate monomers after pretreatment of the tooth and the crown. n= 8	Procera Alumina: CAD/CAM with aluminum oxide coping + Nobelrondo Alumina: feldspathic porcelain powder/liquid	Zinc-phosphate conventional cement; mix and apply RelyX Unicem: hybrid (adhesive resin cement without tooth pretreatment); mix and apply Panavia F 2.0: adhesive bonding (composite resin and pretreatment of tooth and crown); Tooth: ED Primer A+B; Crown: Airborne-particle abrasion Al_2_O_3_, primer and porcelain bond activator; cement: mix and apply	Human teeth	Anatomic crown	1.2 million cycles; 1.6 Hz; 50 N; 8-mm diameter ceramic ball piston; wet.	Fracture load (N)	Compression; no piston reported; 1 mm/min
Al-Wahadni et al, 2009	IPS Empress 2/GIC; In-Ceram/GIC; IPS Empress 2/resin cement; In-Ceram/resin cement. n= 10	IPS Empress 2: pressable lithium disilicate In-Ceram	GIC (Universal Glass Ionomer): p/l 1:2 resin cement (Illusion Universal Cementation System): tooth: H_3_PO_4_ 32%, 15 s. Bonding agent: two coats, light curing per surface. Crown: sandblasting with Al_2_O_3_ + 4% HF, 4 min + silane, 30 s + light-cured paste, light curing 60 s	Human teeth	Anatomic crown	Absent	Fracture load (N)	Compression; 3 mm-diameter ball applied at 45° at 10 mm/min
Rosentritt et al, 2011	Zirconia (Ceramill; Vita YZ Cube; Cercon); glass-infiltrated zirconia (Vita zirconia) X adhesive bonding; conventional cementation. n= 8	Zirconia (Ceramill; Vita YZ Cube; Cercon); glass-infiltrated zirconia (Vita zirconia)	Dual-curing composite, Variolink II; zinc oxide–phosphate cement	Human teeth	Anatomic bridge	1,200,000 mechanical loading cycles of 50 N and 6000 thermocycles for 2 min with distilled water between 5°C and 55°C)	Fracture load (N)	Compression with a 12-mm steel ball at 1 mm/min until fracture
May et al, 2012	Bonded (50, 100, 300, 500 µm), not bonded (50, 100, 300, 500 µm). n=6	Feldspathic porcelain (Vita Mark II blocks)	9% hydorfluoridric acid for 60 s. Primer A and B (Ivoclar Vivadent). Bonded Group silanized (ultradent). For non-bonded groups, poly(dimethylsiloxane) (PDMS). The goups were cemented with Multilink Automix (Ivoclar Vivadent) resin cement	Glass-fiber–filled epoxy resin	Simplified crowns	96-h water storage at room temperature	Fracture load (N)	
Rungruanganun and Kelly, 2012	Panavia/ Zn phosphate; as finished/sandblasted; stored for 14 days. n= 15 Panavia/ Zn phosphate; as finished/sandblasted; stored for 180 days. n= 20	In-Ceram alumina tabs + veneering porcelain: VM7 window porcelain	Half of the tabs (n = 70) were sandblasted with 50-µm Al_2_O_3_ at 2.5 bar pressure (14 s) at a distance of 10 mm. The other half stayed as finished. Zinc-phosphate cement	Woven glass-fiber–filled epoxy; NEMA G10	Simplified restorations	14 or 180 days water storage	Fatigue test under cyclic loading (N) Staircase	Disks were centrally loaded using a 3-mm diameter piston. A sheet of polyethylene (0.1 mm thick) was placed between the piston and disk. At a frequency of 20 Hz, from 10 N to the target load, for 500,000 cycles. The staircase sensitivity method was chosen for the work. Step size was set at 25 N.
Schmitter et al, 2013	Teeth restored with alumina coping and different cements. n=24	Milled alumina	The teeth were treated with 34.5% phosphoric acid, Solobond-Plusprimer, and Solobond-Plus-adhesive, the cores were built up with a self-curing composite. Then the teeth were prepared and the copings were milled. The inner surface of copings was airborne-particle abraded (50-µm aluminum oxide). The ferrule design area of the teeth was etched with 34.5% phosphoric acid for 5 s. 3 cements: a classical glass-ionomer cement (Ketac-Cem), a self-adhesive resin cement (RelyX Unicem) and a conventional resin cement (Panavia F2.0)	Human teeth	Anatomic crowns	Thermal cycles: 10,000 cycles from 6.5 to 60°C (dwell time 90 s, intermediate pause 4 s). Mechanical aging: chewing simulator (1.2 million cycles,maximum force magnitude Fmax=64 N; water storage)	Load to failure (N)	Universal testing machine (crosshead speed of 0.5 mm/min) Universal Pruef Maschine, Z005; Zwick, Ulm, Germany). Loads were applied to the standardized occlusal area (2 mm high, 2 mm wide) at an angle of 45° toward the buccal side of the tooth
Preis et al, 2015	(LDS/ ZLS) Syntac classic/Variolink II (ZLS) Smart Cem 2 (ZLS) Aqua Cem (ZLS) Ketac Cem n= 8	Zirconia-reinforced lithium silicate (Celtra Duo): CAD/CAM block, n= 32 Lithium disilicate (IPS e.max CAD): CAD/CAM block, n= 8 (control)	5% HF 20s (LDS) and 30s (ZLS) (LDS/ ZLS) Syntac classic/Variolink II: adhesive. Silane 60 s on crown + Syntac Classic on tooth + cement application. (ZLS) Smart Cem 2: self-adhesive cement (ZLS) Aqua Cem/Ketac Cem: GIC (ZLS) Ketac Cem: GIC	Human teeth	Anatomic crowns	1,200,000 cycles; 1.66 Hz; 50 N; human molar antagonists + 3000 thermal cycles	Fracture load (kgf)	Compression; 12-mm–diameter ball; 1 mm/min
Campos et al, 2016	Group ZP: no zirconia surface treatment + zinc- phosphate cement. Group PN: no zirconia surface treatment + resin cement. Group AL: air abrasion with alumina particles (125 µm) + resin cement. Group CJ: air abrasion with alumina coated with silica particles (30 µm) + silane + resin cement. Group GL: application of a glaze layer + etching with hydrofluoric acid + silane + resin cement. n= 15	Zirconia blocks - Vita InCeram 2000 YZ	Zinc-phosphate cement Preparations: ultrasonically cleaned in distilled water for 5 min + cement application Dual-activated resin cement (Panavia F) Preparations: 9% HF 1 min, ultrasonically cleaned in distilled water for 5 min + silane (Clearfil Porcelain Bond Activator þ Clearfil SE Bond Primer) + adhesive system (ED primer) + air stream 60 s + cement application	Glass-fiber–filled epoxy resin	Simplified posterior full crowns	Absent	Stepwise stress fatigue test (N)	Stepwise stress fatigue test. In each step of 10,000 cycles, a load of 600 to 1400 N (200-N increments) was applied, with a frequency of 1.4 Hz, in an aqueous environment. The load was applied by means of a stainless-steel piston ball 40 mm in diameter
de Kok et al, 2017	POLISHED SURFACE (Bonding (static test/ Fatigue), simulated (static/fatigue test), Control (static/fatigue test); ROUGH SURFACE (bonding (static test/ fatigue), simulated (static/fatigue test), control (static/fatigue test). Static test n=10. Fatigue test n=20	Lithium disilicate: IPS-Emax-CAD (Ivoclar Vivadent)	Bonding procedure: Substrates were air abraded with 50µm aluminum oxide for 10 s, 38% phosphoric acid conditioning for 20 s. Scotch Bond Universal adhesive (3M Oral Care) was applied. Ceramic was etched with 9.5% hydrofluoric acid for 20 s, primer (Clearfield Ceramic Primer). No bonding procedure: after all bonding treatments, a thin layer of paraffin oil was applied over the epoxy resin, aiming to avoid bonding between ceramic and substrate, followed by cement application, following all steps previously described.	Glass fiber–filled epoxy resin	Simplified restorations	Absent	Step stress fatigue test (N)	Compression; 4.9-mm-diameter ball; 1 mm/min
Sahin et al, 2018	A1 (bioactive cement), B1 (resin cement), C1 (glass-ionomer cement), and D1 (resin-modified GIC) were subjected to a chewing simulation test with thermocycling and mechanical loading (CSTTML). Groups A2 (bioactive cement), B2 (resin cement), C2 (GIC), and D2 (resin-modified GIC) were not subjected to CSTTML. n= 15	Preformed pediatric zirconia crown NuSmile	Bioactive cement, NuSmile. Apply on crown and put in position. G-CEM LinkForce: dual-curing adhesive resin cement. G-Premio BOND on tooth + cement Fuji One: GIC Fujicem 2: resin-modified GIC	Primary molars with enamel caries and an intact crown	Preformed pediatric crown	250,000 cycles of chewing simulation; 50 N; 5-mm stainless-steel ball + 250,000 thermocycles	Fracture load (N)	Compression; piston dimensions not stated; 0.5 mm/min
Vohra et al, 2020	Dentin bonded all-ceramic crowns luted with Bioactive, resin and glass-ionomer cements. n=10	Lithium disilicate: IPS-Emax-CAD (Ivoclar Vivadent). Milled	9.5% hydroﬂuoric acid for 30 s. Single application of silane (Monobond S, Ivoclar Vivadent). Group 1 (n=20): Bioactive (Activa Bioactive cement, ACTIVA, Pulpdent); Group 2 (n=20): resin (positive control) (Nexus 3, Third Generation, Kerr); Group 3 (n=20): glass-ionomer cement (negative control) (GIC Ketac Cem Maxicap, 3M Oral Care)	Human teeth	Anatomic crowns	One half: none The other half of the samples in each cement group were thermocycled (50,000 cycles) between 5°C and 55°C water baths (dwell time 30 s)	Failure load (N)	Compression with a round-head stainless steel probe contacting both lingual and buccal cusp slopes at a crosshead speed of 1 mm/min until failure

Regarding the tests performed, two studies used the step-stress fatigue approach, while the majority^18^ conducted load-to-failure monotonic tests. Furthermore, twelve articles employed an aging strategy, among which most used thermocycling, mechanical fatigue, or a combination of the two.

### Meta-Analysis

A meta-analysis was performed with 21 data sets, although 20 studies were included in the analysis, because one study presented one data set for anterior crowns and one for posterior crowns.^37^ A total of 63 comparison sets were considered in the overall analysis, as a large number of studies had multiple interest groups within the data set, such as different ceramics, cements, and aging conditions.

**Fig 2:**
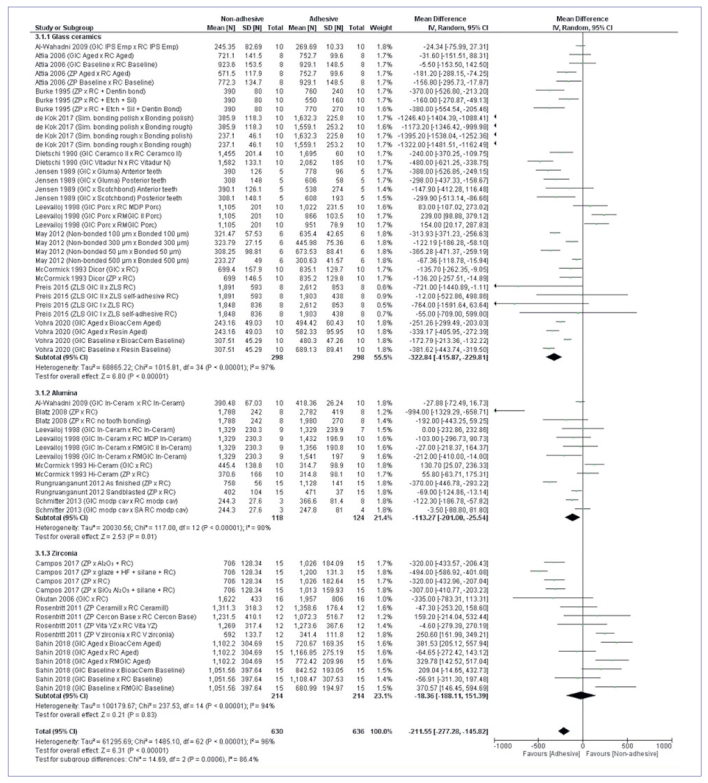
Meta-analysis illustrating the ceramic composition subgroup.

**Fig 3:**
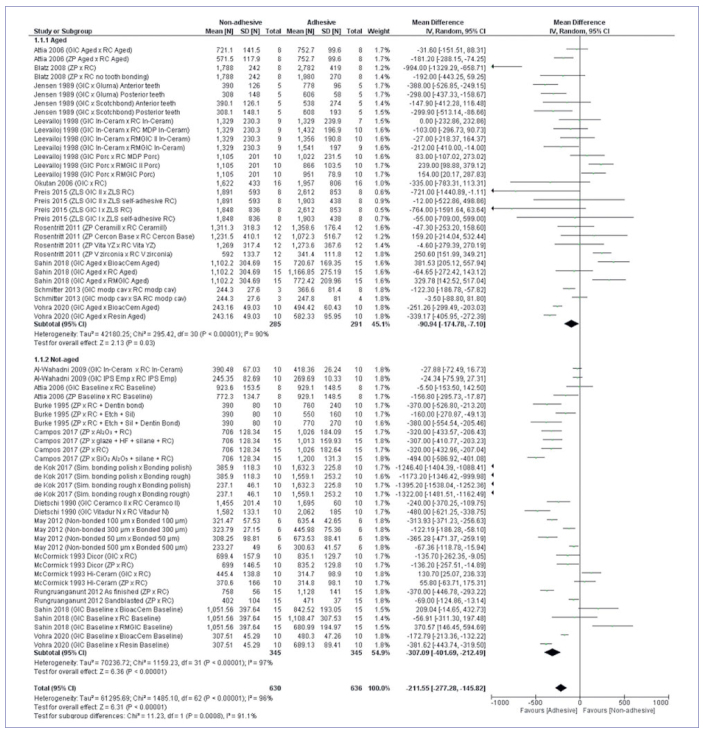
Meta-analysis illustrating the aging condition subgroup.

Considering the overall analysis, dental ceramics that used adhesive luting presented superior in vitro mechanical properties (mean difference of 211.55N CI 95% -277.288, -14582; p ≤ 0.01). Glass ceramics showed favorable results with adhesive luting in the subgroup analysis (Fig 2) considering the ceramic structures (p ≤ 0.01). The alumina subgroup also showed favorable results towards adhesive luting (p ≤ 0.01). The zirconia subgroup showed no difference between adhesive and non-adhesive luting (p = 0.83). In the subgroup analysis considering aging (Fig 3), both aged and non-aged groups presented higher values for adhesive luting (p ≤ 0.01).

### Risk of Bias

**Fig 4:**
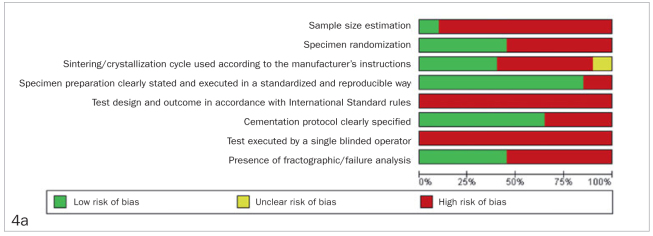

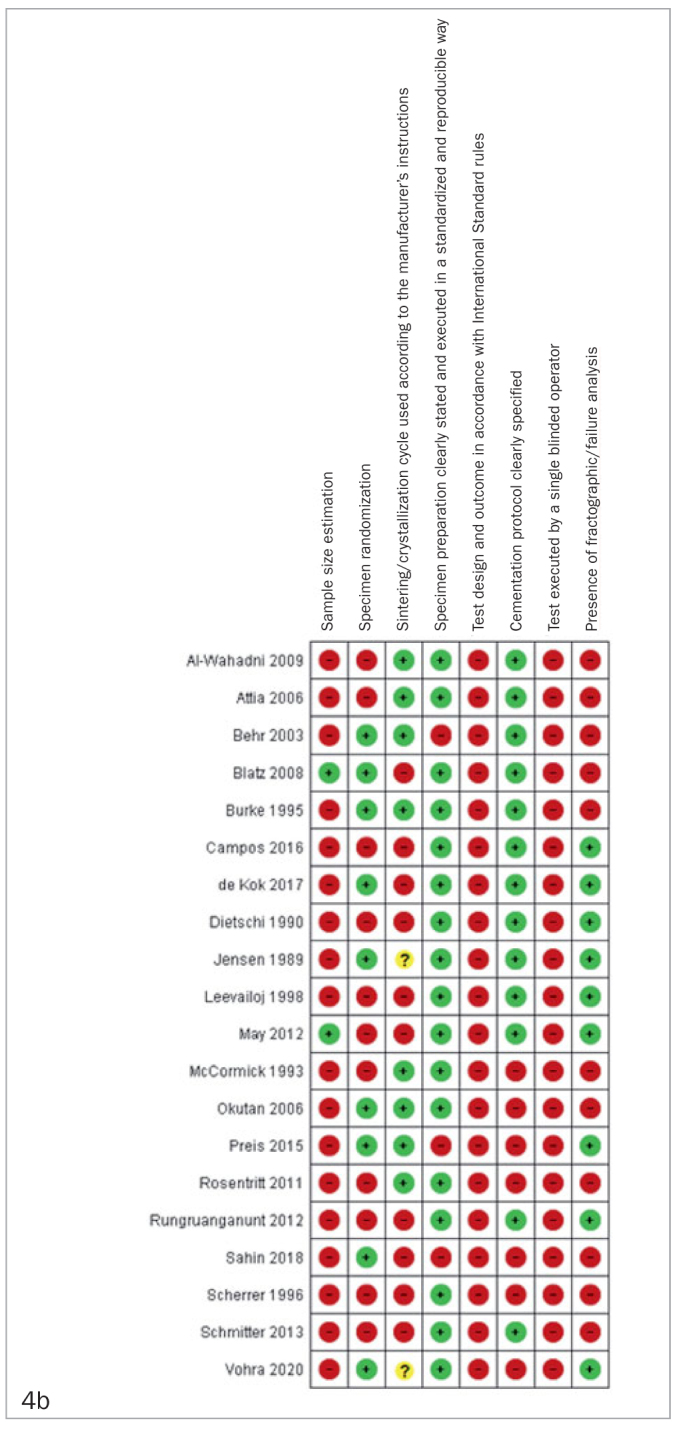
Results of risk of bias analysis.

The results are described in Fig 4 according to the parameters considered in the analysis. All analyzed studies scored negatively for most of the verified bias items. No item received positive scores from all studies.

## Discussion

Adhesive luting improves the mechanical properties of ceramic restorations, except for those made of zirconia. Despite the undeniable importance of adhesion on the fundamentals of restorative dentistry today, the present systematic review and meta-analysis is the first to assemble in vitro data about adhesive luting as reinforcement for restorative dental ceramic mechanical properties. This is an important work which furthers understanding the relation between ceramic, cement, and stress distribution in indirect restorations, thus lending support to clinical decision-making with the best evidence-based practice.

Based on the meta-analysis results (Figs 2 and 3), it can be inferred that adhesive luting reinforces the mechanical properties of dental ceramics used as restorative materials. This can be explained by the differences in cement composition and how they interact with the restoration and the substrate. The non-adhesive materials used were zinc-phosphate (ZP) and glass-ionomer (GI) cements. The first consists in an amorphous matrix of zinc aluminophosphate filled with unreacted zinc oxide particles and does not chemically bond to either dental tissues or restoration; its “bonding effect” is strictly based on mechanical interlocking.^6,35^ The second is a combination of undissolved glass particles coated with silica gel embedded in an amorphous matrix of hydrated calcium and aluminum polysalts containing fluoride.^6^ GI cement is known for its chemical adhesion to the calcium present in dental tissues; however, it also relies on mechanical principles when it comes to restoration retention.^29,90^ It must be mentioned that this kind of cement may vary its properties depending on the powder:liquid ratio, temperature, and moisture during the mixing procedure.^35^ Excess moisture during cementation leads to higher cement solubility, especially at the restoration margins, while a dry condition leads to microcracks.^35^

Resin-modified glass-ionomer cements consist of an aqueous solution containing polyacrylic acid, hydroxyethylmethacrylate (HEMA), and a methacrylate modified polyacrylic acid, as well as a powder containing fluoroaluminosilicate glass, similar to traditional GI cement, together with photo- and chemical initiators.^42^ The polymerization process is important for the initial setting and lowers the solubility of the material compared to conventional GI cement; however, the acid-base reaction is still its main setting mechanism.^35^ On the other hand, the presence of resin monomers, even in small portions, enables this material to have some interaction with the silane applied on glass ceramics, indicating some level of adhesion,^52^ although HEMA is a highly hydrophilic monomer with a reduced degree of conversion and mechanical properties.^31^ In the present meta-analysis, this cement was considered as an adhesive, and by that it is important to note that its use enhances the ceramic restoration’s properties in comparison to non-adhesive cements (ZP and GI).

Alternatively, the most common “adhesive” cementation is based on the use of resin cements, which share the same basic composition, but may differ in application mode (with or without adhesives and etching, depending on the strategy and restorative material).^13,23,38,64,76^ The structure of resin cements is quite similar to that of the resin composites,^7,8,21,31,32,36,78^ along with the inclusion of fillers, such as ceramic particles and colloidal silica, ranging from 40% to 60% by weight.^3^ Some resin cements contain organophosphates such as 10-methacryloyloxydecamethylene phosphoric acid (10-MDP). The viscosity of the resin cements is mainly related to its filler portion, which also influences the elastic modulus and hardness.^32^ These properties are crucial for the restoration’s mechanical behavior, as cements with higher elastic moduli foster better stress transfer from the restoration to the teeth.^63,74^ The filler content is also responsible for the cement viscosity, which is responsible for filling the gaps generated due to surface treatments.^10,77^ Such aspects (variations in composition, viscosity, elastic moduli, etc) were not scrutinized in the present meta-analysis. Thus, future studies may help to explore such differences and determine whether these characteristics will impact the mechanical properties of the set.

In relation to microstructure, dental ceramics can be divided into two main classes: glass and polycrystalline ceramics. Adhesion to glass ceramics has been widely studied over the years;^50^ its classic protocol consists of acid etching with 5%-10% hydrofluoric acid (HF) and subsequent coupling agent application, with silane in the form of 3-methacryloxypropyltrimethoxysilane (MPS) being the most commonly utilized.^13,26,48^ HF acid acts by partially dissolving the glassy matrix of the ceramic, generating irregularities, and providing a greater area for adhesion,^67,82,84^ while silane acts as a link, bonding the ceramic’s silica network to the cement’s organic molecules via siloxane bonds.^52^ The resin cement penetrates into the retentions provided by the HF together with the silane coupling agent, providing enhanced stability of the bonded prosthesis and filling cracks which would concentrate stresses in the restoration.^15,65,67,83^

In contrast, the adhesive luting approach in polycrystalline ceramics, especially zirconia, differs from that of glassy ceramics.^67,82,84^ Air abrasion with aluminum oxide is used for this class of ceramics to create irregularities for subsequent cement penetration,^18^ but it is important to note that this procedure may also damage the ceramic surface, potentially initiating cracks.^86^ In this case, silane is not an agent to be used directly on the treated ceramic, as there is no silica available to which this substance can bond.^52,80^ Some techniques use air abrasion with silica-coated aluminum oxide as a surface treatment for polycrystalline ceramics; this results in silica deposition on the ceramic, which allows subsequent use of silane for the adhesion of a resin cement.^14,18^ The use of 10-MDP primers is especially indicated for zirconia ceramics, since this substance is able to chemically bond to zirconia via ionic interactions and hydrogen bonding.^56^ The use of 10-MDP materials appears to provide strong, stable adhesion between this class of ceramic and resin cements.^27,43,47,54^ Nevertheless, the majority of studies that investigated zirconia ceramics did not find significant differences between adhesive and non-adhesive luting;^57,68,70^ it is noteworthy that only a few published studies exist in this context. There are also contradictory findings depending on the method used to test such studies. Although two studies using monotonic tests showed an absence of a mechanical effect using zirconia and a 10-MDP containing bonding agent,^57,68^ one study, using a fatigue approach, resulted in superior mechanical properties.^19^ Thus, more studies are required, especially using methodologies that promote degradation of the interface between restoration and substrate (ie, aging and fatigue).

Regarding aging, the main method used by the studies included in this review was thermocycling, which usually cycles the specimen between 5ºC and 55ºC in water and is considered a clinically relevant method for aging.^49^ In addition to the effect of the temperature, the presence of hydrophilic monomers in resin cements promotes hydrolytic degradation at the interfaces.^16^ Water sorption by resin-based materials may lead to an increase in the modulus of elasticity,^16^ which causes an unfavorable tension distribution in the restoration; more similar moduli of elasticity between cement and restoration lead to better stress distribution, strength, and performance.^2,28^ Self-adhesive resin cements contain a higher quantity of hydrophilic monomers than do other resin cements, being more susceptible to hydrolytic degradation.^33,44^ Even with these issues, resin cements are less prone to degradation in water than conventional acid-base cements, and are able to maintain their properties for longer periods of time.^11^ Thus, these observations point to the necessity of including aging in future pertinent sutides, in order to characterize the long-term performance of the tooth-restoration unit.

The present systematic review has some limitations. The studies included in this review presented an I^2^=96%, indicating high heterogeneity among the reports. It is a challenge to perform a systematic review based on a large variety of cements and ceramics which have been introduced over the years. This review analyzed articles from 1989 to 2020, during which the luting agents changed, as well as the way their modes of application; the methods employed to test mechanical properties have also evolved. Another important limitation concerns the risk of bias analysis, which presented high negative scores for the majority of items, indicating a lack of compliance with fundamental research principles, greatly impacting the validity/reliability of the data.^24,73^ We emphasize the need for greater attention to methodological rigor when conducting and reporting studies, as well as the need for more studies that focus on aging and fatigue methods in this context. In addition, the search was limited to English, but we believe the number of new studies in other languages would be minimal and would not change our findings.

The present systematic review analyzed in vitro studies; its implications for the clinical context should be carefully considered, since laboratory environments differ from the complex environment in the mouth. On the other hand, the authors emphasize that laboratory research is very well suited to isolating the factors focussed on and providing results which can predict the behavior of restorative materials in clinical practice.^39^ It is important for authors to use those methods which more closely simulate clinical reality to provide more clinically relevant information. This would include fatigue approaches^40^ and aging protocols, which are indispensable for the adhesive context.^81^ More studies should be conducted on zirconia substrates, as their mechanical enhancement mechanisms are not yet fully explored, such as the role of progressive loss of adhesion.

## Conclusions

The in vitro literature indicates that adhesive luting reinforces the mechanical properties of dental ceramics used as restorative materials. The exception is zirconia, for which studies on this theme are scarce.

## Clinical relevance:

The use of adhesive luting reinforces the mechanical properties of dental ceramics used as restorative materials, except for zirconia polycrystals.
